# Multifocal High-Grade Pancreatic Precursor Lesions: A Case Series and Management Recommendations

**DOI:** 10.1089/pancan.2019.0001

**Published:** 2019-04-29

**Authors:** Mazhar Soufi, Michele T. Yip-Schneider, Rosalie A. Carr, Alexandra M. Roch, Howard H. Wu, Christian Max Schmidt

**Affiliations:** ^1^ Department of Surgery, Indiana University School of Medicine, Indianapolis, Indiana.; ^2^ Indiana University Health, Pancreatic Cyst and Cancer Early Detection Center, Indianapolis, Indiana.; ^3^ Indiana University Cancer Center, Indianapolis, Indiana.; ^4^ Department of Pathology, Indiana University School of Medicine, Indianapolis, Indiana.; ^5^ Department of Biochemistry/Molecular Biology, Indiana University School of Medicine, Indianapolis, Indiana.; ^6^ Walther Oncology Center, Indianapolis, Indiana.

**Keywords:** IPMN, pancreatic multifocal high-grade dysplastic lesions, pancreatitis, PanIN-3, recurrence

## Abstract

**Background:** The risk of developing invasive cancer in the remnant pancreas after resection of multifocal high-grade pancreatic precursor lesions is not well known. We report three patients who were followed up after resection of multifocal high-grade pancreatic intraepithelial neoplasia (PanIN)-3 or intraductal papillary mucinous neoplasia (IPMN), two of whom eventually developed invasive carcinoma.

**Presentation:** 1) 68-year-old woman who had a laparoscopic distal pancreatectomy for multifocal mixed-type IPMN, identified as high-grade on final pathology, with negative surgical margins. During semiannual monitoring, eight years from the first surgery, the patient developed suspicious features prompting surgical resection of the body with final pathology revealing invasive ductal adenocarcinoma in the setting of IPMN. 2) 48-year-old woman who had a distal pancreatectomy for severe acute/chronic symptomatic pancreatitis, with final pathology revealing multifocal high-grade PanIN-3, with negative surgical margins. Despite semiannual monitoring, two years from the first surgery, the patient developed pancreatic adenocarcinoma with liver metastasis. 3) 55-year-old woman who had a Whipple procedure for symptomatic chronic pancreatitis, with multifocal PanIN-3 on final pathology. The patient underwent completion pancreatectomy due to symptomatology and her high-risk profile, with final pathology confirming multifocal PanIN-3.

**Conclusion:** Multifocal high-grade dysplastic lesions of the pancreas might benefit from surgical resection.

## Introduction

Pancreatic cancer develops from precursor lesions. It is theorized that such lesions progress in a step-wise manner from low grade to high grade to invasive adenocarcinoma. Pancreatic intraepithelial neoplasia (PanIN) and intraductal papillary mucinous neoplasia (IPMN) are two types of precursor lesions. Although identical at the microscopic level, they differ is size, genetics, and detectability with better imaging.

The risk of recurrence after resection of IPMN is generally considered to be low. Approximately 20% of patients will develop recurrence in the pancreatic remnant with a mean follow-up of 66 months.^1^ Some studies have focused on the risk of high grade or invasive IPMN recurrence after resection of a unifocal high-grade dysplastic lesion,^1,2^ but to our knowledge, there have been no published reports investigating the effect of multifocal high-grade dysplasia on recurrence. In this study, we present three such cases to increase physician awareness regarding the possibility of recurrence and the need for surveillance.

## Case 1

**FIG. 1.**
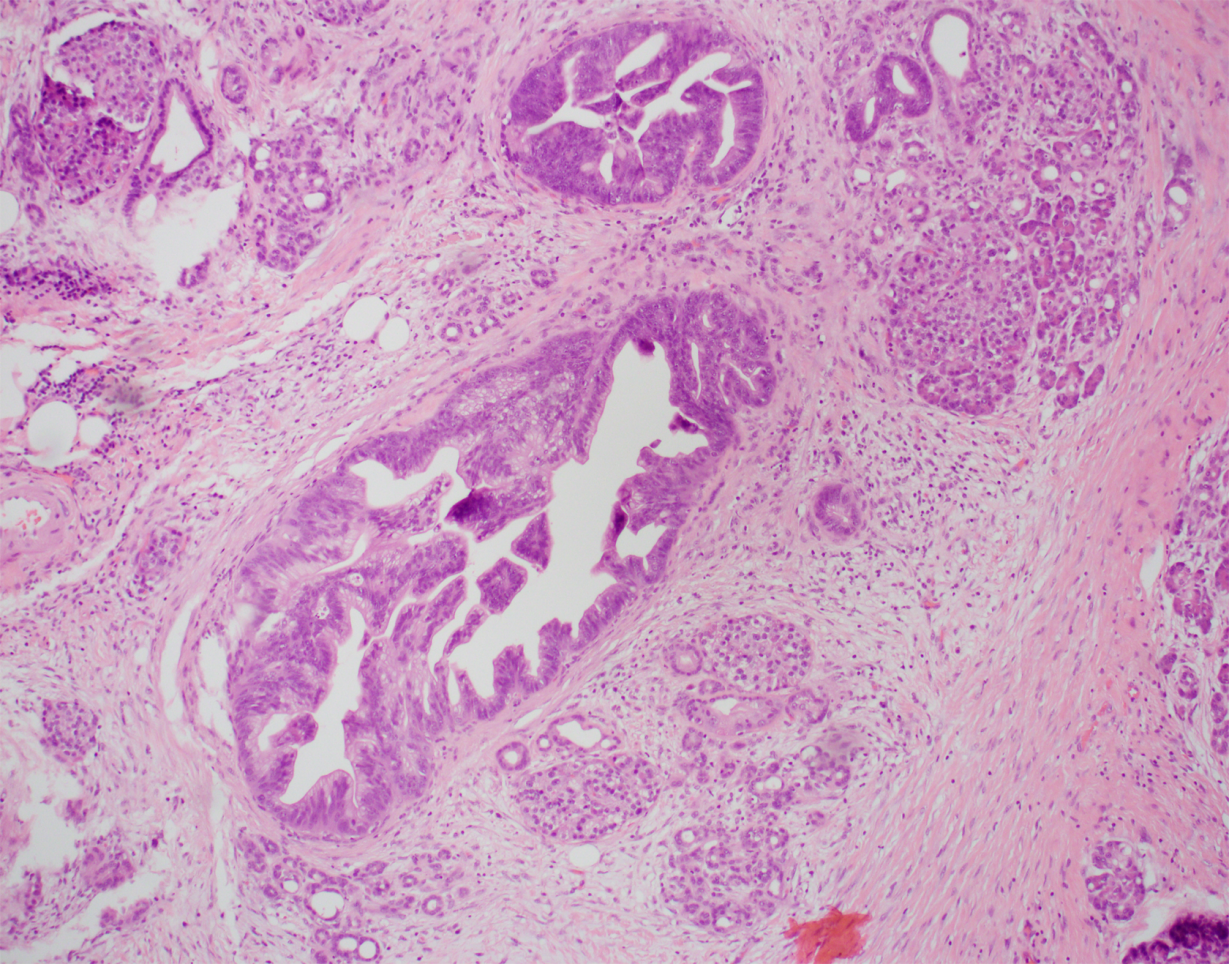
H&E ×100 specimen of pancreatic tail displaying multifocal high-grade intraductal papillary mucinous neoplasia. H&E, hematoxylin and eosin.

**FIG. 2.**
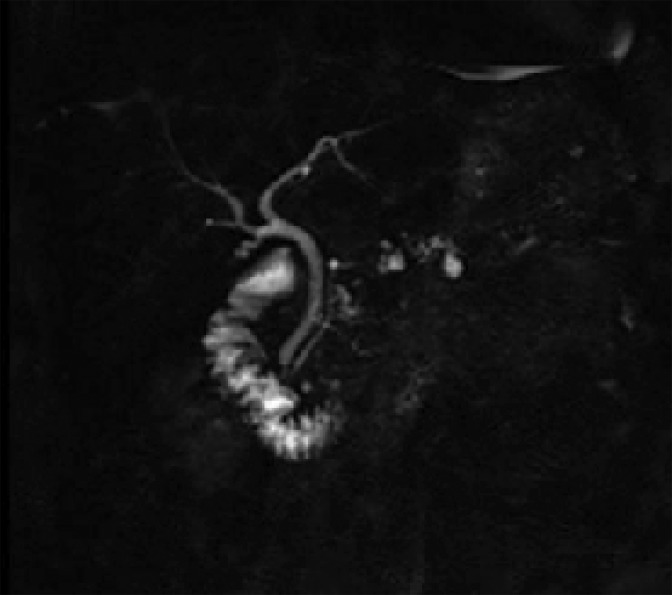
Magnetic resonance cholangiopancreatography demonstrating a new development of enhancement of a branch duct cystic lesion most distally.

A 68-year-old woman had a laparoscopic distal pancreatectomy for multifocal IPMN-mixed type with two foci of high-grade dysplasia detected on final pathology analysis (Fig. 1). Surgical margins were negative. The patient's remnant pancreas continued to be monitored semiannually through cross-sectional studies and intermittent endoscopic ultrasound (EUS) and fine-needle aspiration (FNA). Eight years from the date of the first surgery, the patient was still symptomatic with intermittent twinges of discomfort in her left upper abdominal quadrant. Although her physical examination was unremarkable, her work-up did reveal an elevation in CA19-9 level from 35 to 44 μ/mL. Magnetic resonance cholangiopancreatography (MRCP) disclosed a newly developed internal enhancement of a 14 mm branch duct cystic dilation adjacent to the distal end (Fig. 2). Upper endoscopy with esophagogastroduodenoscopy/FNA of the pancreatic cyst yielded cells consistent with high-grade atypia. She underwent surgical resection of the neck/body of the pancreas, and surgical pathology analysis revealed an invasive well-differentiated adenocarcinoma (stage 1B) of mixed-type IPMN with evidence of chronic pancreatitis. The patient completed 6 months of gemcitabine adjuvant chemotherapy and is still alive 4 years from the time of the second surgery. She was offered completion pancreatectomy but deferred.

## Case 2

**FIG. 3.**
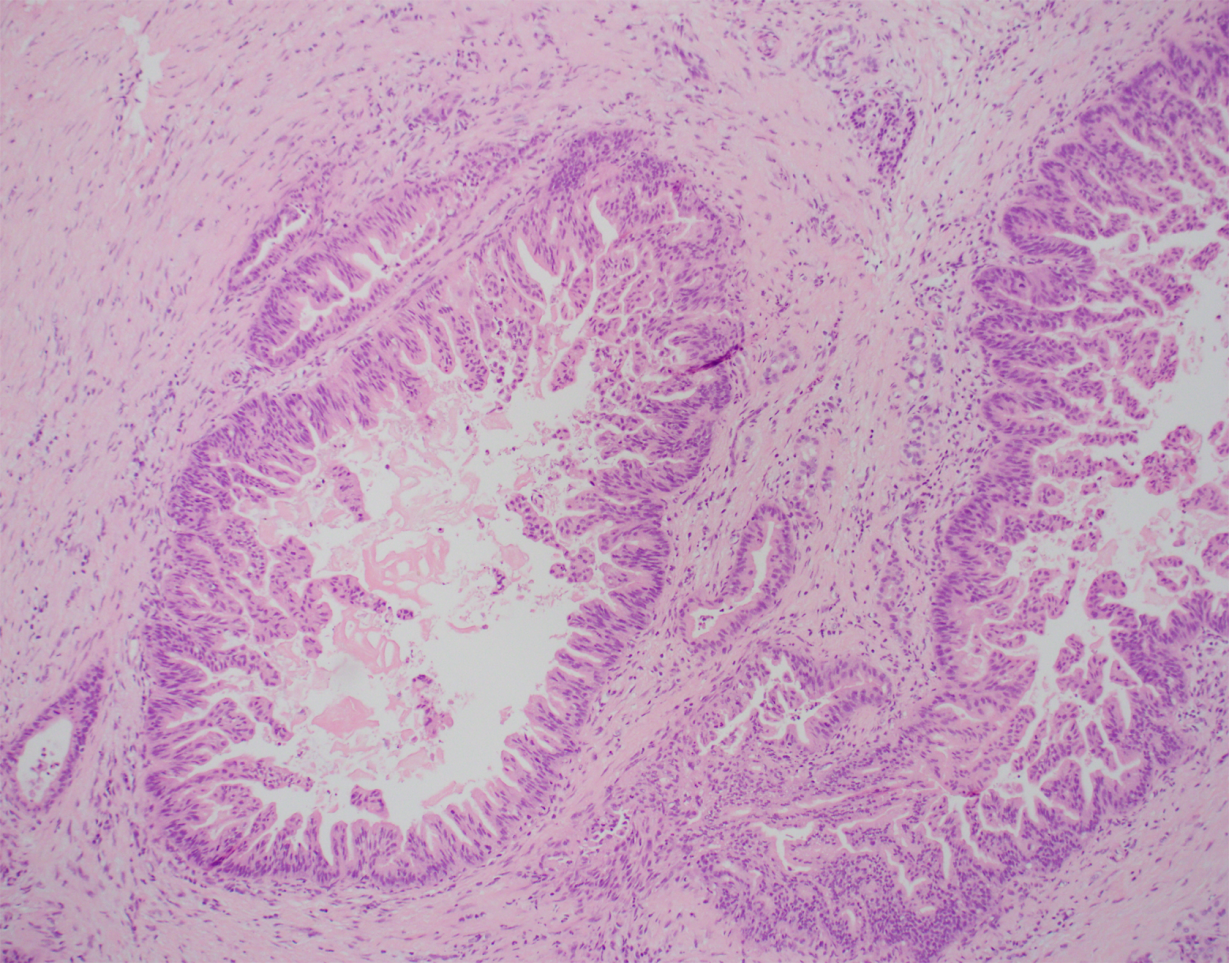
H&E ×100 specimen of pancreatic tail displaying multifocal high-grade dysplasia pancreatic intraepithelial neoplasia-3.

A 49-year-old woman presented with moderately severe pancreatitis. She required three hospitalizations. This was traumatic in etiology as she was kicked by a horse. The patient experienced multiple complications including thromboembolic events and a pancreaticopleural fistula. Owing to escalation of symptoms and failure to thrive, she presented to medical attention, and a distal pancreatectomy was pursued. Surgical pathology analysis revealed multifocal high-grade dysplasia PanIN-3, with evidence of chronic pancreatitis in the specimen (Fig. 3). Three foci of high-grade dysplasia were observed; surgical margins were negative. Based on remembering Case 1, a multidisciplinary team was assembled, which included consultation with a world-renowned pancreatic pathologist. Completion pancreatectomy was offered, but the consultant and team's recommendations were close surveillance. Semiannual surveillance ensued. Unbeknownst to her physicians, the patient developed symptoms of pancreatitis within 6 months of her initial surgery but the patient did not complain or present to medical attention. The patient attributed her symptoms (i.e., back pain) to her occupation. Two years from the time of surgery, a surveillance computed tomography (CT) scan disclosed a newly developed low-density lesion in the head of the pancreas measuring ∼1 cm as well as an isolated liver metastasis; the two lesions were proven to be adenocarcinoma on biopsy.

## Case 3

A 55-year-old woman was referred to our clinic for further management after having had a Whipple procedure elsewhere for chronic pancreatitis. The specimen demonstrated multifocal PanIN-3 with evidence of chronic pancreatitis in the background. Four foci of high-grade dysplasia were observed; surgical margins were negative. The patient was symptomatic with intermittent epigastric pain radiating to the back, which was thought to be related to pancreatitis. The pain was severe enough to affect her daily activities, resulting in a chronic narcotic-dependent status and hospitalizations for pain control. Recent outside CT imaging confirmed changes compatible with chronic pancreatitis in the remnant pancreas, as well as the presence of a retained pancreatic stent thought to be partially contributing to her pain. Her family history is significant for pancreatic cancer, her sister developing it in her 60s. Owing to her symptomatology, the retained stent, significant family history, the initial surgical pathology report, and our experience with the mentioned two cases, completion pancreatectomy was recommended and pursued. Final pathology report disclosed the presence of multifocal PanIN-3 in the setting of pancreatitis.

## Discussion

The presence of multifocal high-grade dysplastic lesions raises intriguing questions about pancreatic tumorigenesis. Two possible scenarios may explain the simultaneous occurrence of multiple lesions. First, all the lesions may share a similar genetic or molecular background leading to malignant progression, also known as the field theory; or second, these lesions may arise independently and not share a common tumorigenic pathway or event. In a recently published study aimed at establishing a relationship between histological subtypes of IPMN and frequencies of specific genetic mutations,^3^ Tan et al. examined the mutation status of adjacent areas within the same patient's invasive IPMN specimen. For 28% of the cases, they detected different mutations of the same gene in adjacent areas of varying dysplastic grade from the same patient; moreover, they reported the absence of mutations in specimens of higher levels of dysplasia that were present in specimens of lesser dysplasia from the same patient. These findings support a multifocal nature of the tumorigenic process within the pancreas. The authors propose that a field defect exists in which the entire pancreas may be prone to the accumulation of distinct genetic mutations and errors that may eventually evolve into cancer.

In addition, the presence of chronic pancreatitis in two of our cases highlights the effect that pancreatitis may have on the outcome. In one cohort study, patients with chronic pancreatitis were found to have an increased risk of pancreatic cancer.^4^ Specifically, 1552 subjects were followed up for a minimum of 2 years, and of these, 29 (21 male and 8 female subjects) had evidence of cancer of the pancreas 2 or more years after the diagnosis of pancreatitis during a mean follow-up (±standard deviation) of 7.4 ± 5.6 years. In another case–control study, the relative risk in patients with chronic pancreatitis was found to be as high as 13.3 compared with the control group.^5^

The presence and extent of invasive IPMN have consistently been identified as risk factors for poor outcomes.^2,6^ However, risk factors for local recurrence of noninvasive lesions are not well defined. Furthermore, the risk of recurrence of multifocal high-grade dysplasia has not been studied extensively. Prior study evaluating the association between unifocal high-grade dysplasia and recurrence has shown that the degree of dysplasia seen on the specimen is a risk factor.^2^ The rates of recurrence significantly increased as the grade of dysplasia increases. After a median of 44.4 months of follow-up, 39 out of 366 (10.7%) patients experienced disease recurrence with a median of 18.8 months, and of these 16 (41%) patients did not initially have an invasive IPMN. Analysis of these recurrences shows that six patients with high-grade dysplasia developed recurrence—one of them had an adenocarcinoma, two had recurrent IPMN, and three had distant metastasis. Therefore, thorough postoperative surveillance is needed not only for invasive IPMN but also for benign noninvasive lesions and especially those with high-grade dysplasia.

At the Indiana University Pancreatic Cyst and Cancer Early Detection Center, high-risk patients (i.e., Cases 1 and 2) undergo surveillance that includes annual EUS-FNA as well as semiannual magnetic resonance imaging (MRI)-MRCP. Unfortunately, the surveillance strategy failed for Case 2, prompting a more aggressive approach in managing Case 3, potentially preventing malignant recurrence. Therefore, based upon our experience, we recommend that total pancreatectomy be strongly considered in fit patients with multifocal high-grade lesions, whether or not pancreatitis is present. However, pancreatic surgery is high risk and has a morbidity rate of 45–55% and a mortality rate of 2–4%.^7,8^ Complications after total pancreatectomy may include brittle diabetes, a very challenging form of diabetes that is resistant to insulin treatment, thought to originate from a lack of glucagon secretion as a result of the surgery. The patient may also develop pancreatic exocrine insufficiency with its associated fat-soluble vitamin deficiency as well as malnutrition, typically addressed by adding supplemental pancreatic enzymes.

## Conclusion

Although the surveillance protocol for a resected invasive IPMN is well established, the follow-up for a noninvasive cystic lesion depends on many factors and is still a subject of controversy. The rates of recurrence for unifocal noninvasive IPMN are reported to be ∼20%.^1^ Therefore, the guidelines recommend indefinite close surveillance after achieving clear surgical margins. However, the risk of progression from multifocal high-grade PanIN or IPMN into invasive cancer is unclear, and no clear recommendations for the management of these patients exist to date. Based upon our experience, we are recommending strong consideration of completion pancreatectomy in fit patients. In the event, completion pancreatectomy is not pursued, we would recommend quarterly surveillance with serum markers, MRI-MRCP, and semiannual EUS-FNA.

## Abbreviations Used

CT: computed tomography
EUS: endoscopic ultrasound
FNA: fine-needle aspiration
H&E: hematoxylin and eosin
IPMN: intraductal papillary mucinous neoplasia
MRCP: magnetic resonance cholangiopancreatography
MRI: magnetic resonance imaging
PanIN: pancreatic intraepithelial neoplasia
